# Developing a Revised Short Form of the Practice Environment Scale for Home Healthcare: A Nationwide Cross-Sectional Study for Healthcare Professionals

**DOI:** 10.7759/cureus.97403

**Published:** 2025-11-21

**Authors:** Yasuko Ogata, Miki Sasaki, Ayano Fujiyoshi-Ito, Nobuko Lapreziosa, Yuki Yonekura

**Affiliations:** 1 Graduate School of Health Care Sciences, Institute of Science Tokyo, Tokyo, JPN; 2 Department of Hospice and Palliative Care, St. Luke's University Health Network, Bethlehem, USA; 3 Graduate School of Nursing Science, St. Luke's International University, Tokyo, JPN

**Keywords:** home healthcare, instrument development, intention to remain, job satisfaction, reliability, validity, work environment

## Abstract

Introduction

With staffing shortages, retaining healthcare professionals working in home healthcare is one of the key strategies. Easier tools are needed to capture the work environment characteristics associated with their retention. This study aims to develop a shortened version of the Practice Environment Scale for Home Healthcare, capable of measuring the attractiveness to healthcare professionals.

Methods

A mail survey with anonymous self-administered questionnaires was distributed to healthcare professionals working at 153 (rate of intention to participate: 7.7%) home-visit nursing agencies in Japan (July-October 2023). The questions included 37 items from the original version of the Practice Environment Scale for Home Healthcare, which comprises seven factors, including job satisfaction, quality of care, and intention to remain. Confirmatory factor analysis was conducted on 21 items from 380 (rate of valid responses: 31.4%) mail surveys. The relationships between the subscale and composite scores of the 21 items for the home healthcare practice environment scale and participants’ job satisfaction, quality of care, and intention to remain at the agencies were assessed.

Results

A seven-dimensional model with 21 items was supported by a confirmatory factor analysis. The Cronbach’s alpha of the seven subscales and the composite was 0.80-0.91. The scores on the Practice Environment Scale for Home Healthcare were positively associated with participants’ job satisfaction, quality of care, and intention to remain.

Conclusion

A shortened Practice Environment Scale for Home Healthcare, comprising 21 items, is a valid and reliable measurement that effectively assesses attractive practice environments for healthcare professionals. Improving the characteristics of the work environment, as indicated in the shortened scale, will aid managers and policymakers in securing healthcare professionals in home healthcare settings.

## Introduction

Life expectancy has increased globally, necessitating a more optimized global healthcare system to meet the demands for care. The older population requires assistance with activities of daily living, and those suffering from multiple diseases, in addition to their limited physical mobility, will further increase the demand for long-term care (LTC), especially home care. Further, most older individuals prefer to remain in their own homes, which is also important for their overall well-being. Home healthcare can address the increased demand for healthcare needs worldwide. However, the global workforce shortage in the home care industry remains challenging [1]. This calls for initiatives to retain healthcare professionals in home healthcare settings.

Attracting and retaining workers are crucial in the LTC sector, which faces a significant shortage of healthcare staff [2]. Indeed, the higher the staff turnover rate in LTC nursing homes, the lower the quality of care [3]. Home healthcare staff shortages often result in increased workloads for existing staff, lower job satisfaction, and ultimately, a decline in the quality of patient care. Therefore, enhancing retention and preventing healthcare professional turnover can improve the quality of care. Rewards for healthcare professionals and the nature of work itself vary globally and are influenced by various factors. Therefore, organizational characteristics that attract healthcare professionals must be clarified, regardless of system differences between countries. Specifically, identifying organizational traits that appeal to healthcare professionals and can be enhanced/revised by the initiatives of each organization would be beneficial.

The intention of healthcare professionals or LTC workers to leave or continue working is broadly attributed to two factors: individual or internal factors and organizational or external factors. Individual factors related to intention to stay include sociodemographic characteristics and psychological factors [4]. Job satisfaction is the most influential factor affecting the turnover intention of nurses employed at nursing homes [5]. Organizational factors that influence healthcare professionals’ decisions to leave their positions include job demands, employment services, working conditions, work relationships, and organizational culture [6]. Individual or internal factors, such as age, education level, marital status, and immigration status, are often difficult to change through personal effort or external intervention. On the other hand, organizational or external factors can be improved by altering the way the organization is managed through external intervention or organizational efforts.

Several studies have developed instruments to measure organizational characteristics that attract healthcare professionals [7-9]. In particular, in the United States, from the late 1970s to the 1980s, a period marked by a shortage of nurses, hospitals that attracted nurses and had low turnover rates were designated as “magnet hospitals.” Several instruments were developed to measure the organizational characteristics of these hospitals, including the Nursing Work Index (NWI) [7], Revised Nursing Work Index (NWI-R) [8], and Practice Environment Scale of the Nursing Work Index (PES-NWI) [9]. The PES-NWI has been translated into 24 languages worldwide and used in numerous studies to evaluate nurses’ work environments [10]. Better work environments, as evaluated by the PES-NWI, were associated with improved outcomes for nurses and patients: lower odds of negative nurse outcomes, poor safety or quality ratings, and negative patient outcomes, but higher odds of patient satisfaction [11]. Furthermore, the work environment of nurses, as measured by the PES-NWI, is significantly related to nurses' intention to leave the hospital [11,12]. The PES-NWI scores not only directly affect nurses' actual turnover but also prevent nurses with high levels of psychological distress from leaving the hospital by reducing psychological distress [13].

For home healthcare nurses, scales designed for hospital nurses, such as the PES-NWI, are being applied to identify the relationship between work environment and the outcomes of acute hospitalization and discharges to community living [14]. Furthermore, scales reflecting work environment characteristics in home healthcare have been developed based on the PES-NWI [15,16], though the number of items in both scales was not small [15,16]; one had low structural validity [15]. Practically, a shorter version of a highly reliable and valid scale to measure the attractiveness of the work environment remains warranted.

The Nursing Practice Environment Scale for Home Healthcare (NPES-HHC) is a valid and reliable scale [16] developed based on the PES-NWI items. Since the NPES-HHC is associated with the intention to remain employed [16], it may also be associated with actual turnover. A prospective study that considers the NPES-HHC as a factor and actual turnover as an outcome could identify work environment characteristics that contribute to turnover. In other words, the NPES-HHC can serve as a guide to prevent turnover. However, given the length of the NPES-HHC (37 items), a shortened version is needed to reduce the burden on respondents and enable managers, researchers, and policymakers to efficiently grasp the information with less effort. Furthermore, unlike when the scale was originally developed, currently, various therapists as well as nurses work in home healthcare agencies in Japan; hence, a revised version of the scale incorporating these professionals is needed.

In Japan, the government-led LTC insurance system, established in 2000, covers in-home services, facility services, and community-based services. Of the in-home services, “home-visit nursing services” are primarily provided by “home-visit nursing agencies” managed by nurse managers. Home-visit nursing agencies also provide rehabilitation services and employ other healthcare professionals, such as physical therapists, occupational therapists, and speech-language-hearing therapists, besides nurses. Corresponding to the demand, the number of home-visit nursing agencies has increased from 4,693 in FY2001 to 15,055 in FY2024 [17]. The number of nurses and non-nursing therapists at home-visit nursing agencies has increased yearly. As per 2023 data, the number of full-time equivalent employees working at home-visit nursing agencies is 103,314. Nurses make up the largest proportion at 63.7%; other professionals, such as physical therapists, occupational therapists, and speech-language-hearing therapists, comprise 14.8%, 6.4%, and 1.3% of the workforce, respectively [17]. It has been estimated that 120,000 nurses will be needed in home-visit nursing agencies by FY2025; however, a shortage currently exists [17]. In addition to increasing the number of healthcare professionals in home healthcare settings by making home-visit nursing agencies more appealing, strategies aimed at retaining the healthcare professionals already working in this setting are also essential and can help address the global shortage of healthcare professionals in home healthcare settings. In this study, we aimed to develop a reliable and valid shortened version of the Practice Environment Scale to assess home healthcare environments for nurses and non-nurse professionals.

## Materials and methods

Hypothesis

This study was conducted with reference to the COnsensus-based Standards for the selection of health status Measurement INstruments (COSMIN) checklist. To achieve the aims of this study, the following hypotheses were developed: Hypothesis (a): The abbreviated scale has internal consistency for each factor and the entire factor, i.e., a high Cronbach’s alpha coefficient (reliability). Hypothesis (b): The short-form version will retain the same seven subscale structures as the original version (construct validity). Hypothesis (c): A better work environment is associated with better job satisfaction among home healthcare professionals (criterion-related validity). Hypothesis (d): A better work environment is associated with better-quality care provided by home healthcare agencies (criterion-related validity). Hypothesis (e): A better work environment is associated with greater intention among healthcare professionals to remain working in home healthcare settings (criterion-related validity).

**Figure 1:**
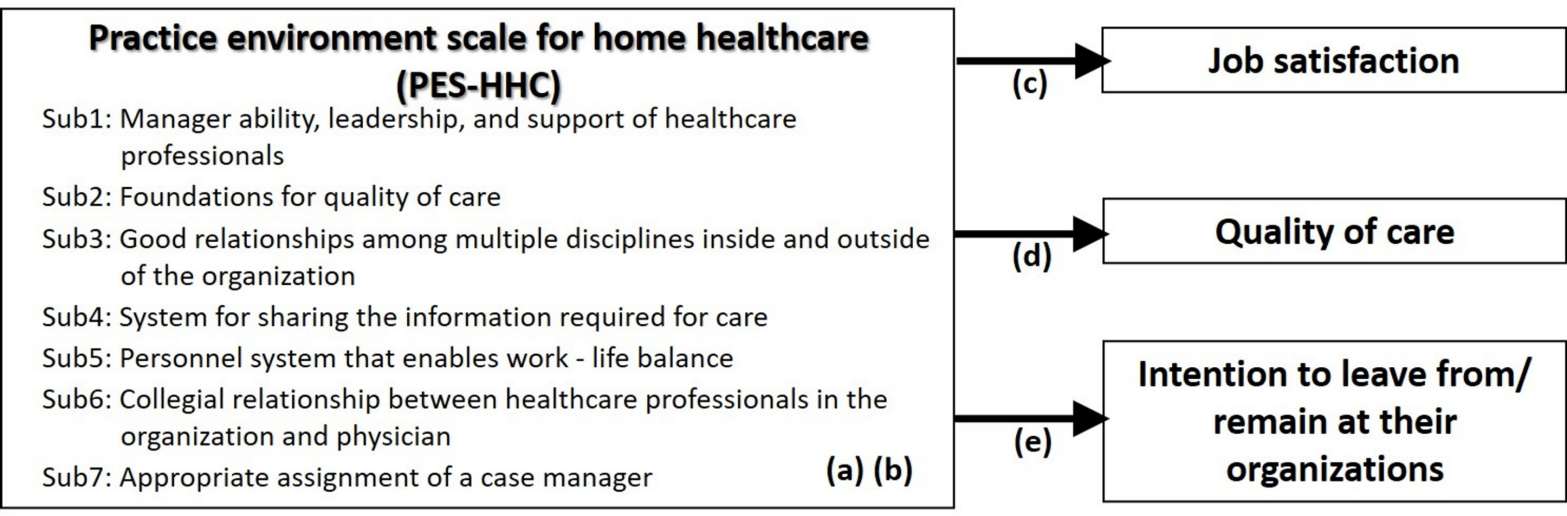
The relationship between PES-HHC and each concept, and the hypotheses Sub1 to Sub7 refer to subscales 1 to 7 of the PES-HHC; (a) to (e) correspond to hypotheses (a) to (e) in "Materials and methods" in the main text. PES-HHC: Practice Environment Scale for Home Healthcare

Figure 1 illustrates the concepts used to test the hypotheses, the relationships among them, and the corresponding hypotheses.

Sampling and recruitment

This cross-sectional study comprised three steps: (1) a self-administered anonymous questionnaire survey was conducted using the original scale (37 items); (2) 21 items, three for each of the seven factors, were selected from the original scale as candidates for the PES-HHC; and (3) the 21 items were analyzed to confirm the reliability and validity of the shortened scale.

**Figure 2:**
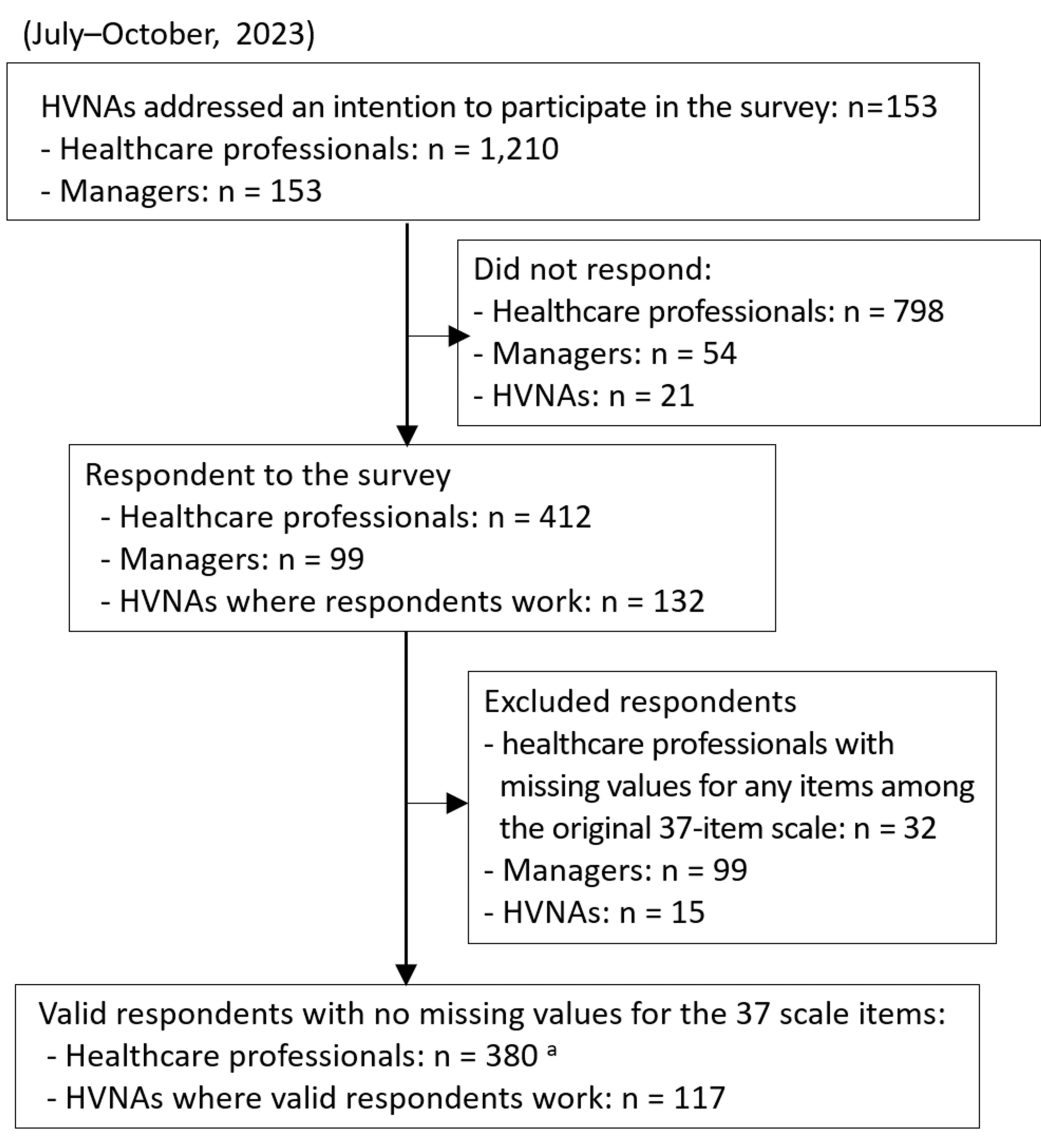
Flow diagram of survey participants ^a^Responses to items other than the 37 scale items include missing values. HVNA, home-visit nursing agency

An anonymous, self-administered questionnaire survey was completed by healthcare professionals, namely nurses, physical therapists, occupational therapists, and speech-language-hearing therapists, working in home-visit nursing agencies in Japan (July-October, 2023). First, from all home-visit nursing agencies nationwide, 2,000 home-visit nursing agencies were randomly selected, stratified by the region in which they were located, and asked to participate in the survey (Figure 2). Of the 2,000 home-visit nursing agencies, 153 (rate of intention to participate: 7.7%) showed a willingness to participate. Second, questionnaires for 1,363 individuals, including managers, were sent to the 153 agencies and distributed to each healthcare professional and manager via the agencies. Responses were received from 412 (rate of response: 34.0%) healthcare professionals and 99 (rate of response: 64.7%) managers between July and October 2023. Data from 380 (rate of valid response: 31.4%) healthcare professional participants with no missing values for the 37 NPES-HHC items were analyzed.

Since the COSMIN study design checklist [18] recommends a sample size of at least seven times the number of items and a minimum of 100 participants, the required number of healthcare professional staff to whom the questionnaire was to be administered was set to at least 1,000, considering the collection rate (assumed to be 30%) and missing data values. Therefore, 1,210 (all healthcare professionals working at facilities that have expressed their intention to participate: 100%) healthcare professionals working in 153 home healthcare agencies were included in this study (Figure 2). To address the potential sources of bias, a stratified random sampling of 2,000 home-visit nursing agencies by region was conducted to ensure that the regions where the participants were located were not biased, and their willingness to cooperate in the study was confirmed. In addition, responses to the questionnaire were anonymized to protect the participants’ identities.

Variables and measurements

The self-administered questionnaire survey included the NPES-HHC, comprising 37 items, as well as job satisfaction, quality of care provided by agencies, intention to remain in the workplace, and participant demographics.

The NPES-HHC measures the attractiveness of the nursing practice environment for home healthcare. It was developed through a three-step process by analyzing mail surveys conducted by nurses at home healthcare agencies, or “home-visit nursing agencies,” in Japan [16]. First, the item pool was created by integrating free text from a mail survey of home healthcare nurses and interview responses from nurses working at home-visit nursing agencies [16]. In the free-form comments and interviews, respondents were asked about the workplace conditions or characteristics that made them want to continue working [16]. Second, with the collaboration of five nurse managers from home-visit nursing agencies, an attempt was made to reduce the item pool based on the five nurse managers’ answers [16]. They were asked to rate each item on a five-point scale in terms of “validity” as an indicator of organizational attractiveness, “feasibility” when attempting to implement it in the organization, and “importance” to determine whether it represents a characteristic of an attractive home healthcare agency [16]. For example, the answer options for appropriateness were “valid (5),” “can't say either way (3),” and “not valid (1)” [16]. Third, a mail questionnaire survey was conducted with nurses from 421 (rate of intention to participate: 7.4%) home-visit nursing agencies. After analyzing 1,050 valid responses (rate of valid response: 35.9%) using exploratory and confirmatory factor analysis, the NPES-HHC, comprising 37 items and seven factors, was developed [16].

In the current survey, all 37 NPES-HHC items were included in the self-administered questionnaire. However, given that the respondents included therapists and nurses, the subject of each question was modified from “nurse” to “healthcare professional.” Accordingly, the name of the revised, shortened scale is “the Practice Environment Scale for Home Healthcare (PES-HHC).” The four response options for the 37 questions were strongly agree (4), agree (3), disagree (2), and strongly disagree (1). Each subscale score was calculated as the average of the responses to the questions on each subscale. The composite score was calculated as the average of the seven subscale scores. The scores ranged from 1 to 4, with 2.5 being the theoretical median. Following the questionnaire survey, 21 of the 37 NPES-HHC items were selected, and confirmatory factor analysis was performed. Since the COSMIN study design checklist [18] recommends confirmatory factor analysis as the first choice to confirm the structural validity of the scale, we employed confirmatory factor analysis in this study.

The seven subscales were also revised to include content about healthcare professionals, including therapists and nurses: “1. Manager ability, leadership, and support of healthcare professionals, 2. Foundations for quality of care, 3. Good relationships among multiple disciplines inside and outside of the organization, 4. System for sharing the information required for care, 5. Personnel system that enables work-life balance, 6. Collegial relationship between healthcare professionals in the organization and physician, and 7. Appropriate assignment of a case manager.”

“Intention to remain” was posed as an original question in the questionnaire. The participants were asked whether they would “stay or leave their current home healthcare agency within the next year”; their responses were on a four-point scale: “working,” “probably working,” “probably not working,” or “not working.” Responses were categorized into two groups: “intention to leave” (“probably not working” or “not working”) and “intention to remain” (“working” or “probably working”).

“Job satisfaction” and “quality of care” were assessed using a visual analog scale (VAS). Respondents, namely healthcare professionals at home-visit nursing agencies, were asked to evaluate their degree of satisfaction with their job and the quality of care they provided to their clients on a 10-cm straight line, with the length in millimeters as the score. Respondents were presented with two 10-cm horizontal lines and asked to draw a vertical line on each horizontal line, where the respondent rated the “degree” of job satisfaction or quality of care as the most indicative. Job satisfaction was assessed by asking, “To what extent are you ‘satisfied’ with your current workplace?” The quality of care was assessed by inquiring, “How would you rate the ‘quality’ of home healthcare provided by this home healthcare agency?” The VAS is a scale used to measure subjective experiences such as pain and fatigue [19] and is also used to measure job satisfaction [16,20-21] and quality of care [16].” Organizational and workforce research has validated the use of VAS to measure job demands and job control, verified by comparing the existing scale with 18 items [22]. Based on these previous studies and the fact that the VAS reflects subjective experiences [19], we decided that the VAS can be used to assess satisfaction and quality of care from the perspective of healthcare professionals regarding their own work experiences. Since there was no data available at the time of the survey on quality indicators commonly used by home care providers in Japan, we decided to use the VAS to assess the quality of care in addition to job satisfaction.

Demographic questions included age, sex, academic background, employment status, profession, and years of professional experience as a nurse, physical therapist, occupational therapist, or speech-language-hearing therapist. To understand the characteristics of the agencies, the participants were also asked about the number of staff members, the number of visits to clients’ homes per month, and the founding entities.

Data analysis

Several steps were taken to develop the shortened version of the Practice Environment Scale. First, the 37 items of the NPES-HHC (an original scale) comprising seven factors were reduced to 21 items, with three items per factor, while ensuring content validity. Second, to confirm internal consistency, Cronbach's alpha coefficient was calculated for the three items comprising each factor. Third, to confirm construct validity, a confirmatory factor analysis was performed using all variables of the seven factors and 21 items according to COSMIN guidelines [18]. Furthermore, to confirm criterion-related validity, the relationship between the seven subscale scores and the composite score of PES-HHC (21-item), as well as job satisfaction, quality of care, and healthcare professionals’ intention to leave or remain in the workplace, was examined. Analyses were performed with IBM® SPSS® Statistics for Windows version 29.0.2.0 (IBM Corp., Armonk, NY) and IBM® SPSS® Amos™ version 30.0.0 (IBM Corp., Armonk, NY).

Validity

To develop a short version of NPES-HHC, three items for each factor were retained based on discussions among nursing researchers and home healthcare expert nurses' judgment. The items of the six subscales, except for subscale 7, were reduced to three items per subscale. As the number of items in subscale 7 was already three, all items were retained.

Three home healthcare expert nurses, one of whom was also the manager of a home healthcare nursing agency, ranked each item from 1 to 5 for each subscale, excluding subscale 7, in order of priority, to obtain the most representative of the subscale's content. That is, they were asked to rank each item from 1 to 5 for each subscale. Based on the assigned rankings, the three nurse researchers discussed and selected three items per subscale to retain the most representative item of that subscale. During this process, three nurse researchers reviewed the rankings provided by three home healthcare expert nurses and selected items based on the following two criteria: (1) items ranked within the top five by at least two of the three home healthcare expert nurses (a mandatory requirement for selection) and (2) items with relatively small rankings (high evaluations) by the three home healthcare expert nurses (a reference criterion for selecting the item). The higher the rating (smaller number) given by the three home healthcare expert nurses, the more appropriately that item represents that subscale. For example, the item “A Clear philosophy of service that pervades the patient care environment” was rated first by Nurse A, first by Nurse B, and second by Nurse C. Thus, it satisfied requirement (1), i.e., “items rated within the top 5 by at least two of the three home healthcare expert nurses.” Furthermore, due to the high ratings (smaller number) from all three experts, this item was selected for the shortened version of the PES-HHC. Thus, to ensure content validity, the researchers selected items based on the judgment of experts who were well-versed in the actual home healthcare situation in Japan. For items on which the three nurse researchers disagreed, the rankings determined by the three home healthcare expert nurses were repeatedly confirmed, with emphasis on (1) and (2), and the three nurse researchers discussed the items until the disagreements were resolved.

Confirmatory factor analysis was performed to confirm construct validity, whether the shortened version of the 21-item scale had the same 7-factor structure as the original version of the 37-item, according to the COSMIN guidelines [18]. Model fit was assessed using the Goodness-of-Fit Index (GFI), Normed Fit Index (NFI), Comparative Fit Index (CFI), and root mean square error of approximation (RMSEA). Using confirmatory factor analysis, the short version of the scale was assessed to confirm that it had the same seven-factor structure as the original version. Through this process, hypothesis (b) was tested, namely, whether “the short-form version retains the same seven subscale structure as the original version.”

Criterion-related validity was confirmed with partial correlation coefficients calculated between the subscales and composite scores of the shortened NPES-HHC and job satisfaction and quality of care after adjusting for age, sex, and education level. Criterion-related validity was confirmed in the same manner as when the original scale was developed. Intention to leave was assessed based on the question “Will you still be working at your current home healthcare agency next year?” Those who answered “(probably) still working” were considered to have the intention to remain, while those who answered “(probably) not still working” were considered to have the intention to leave. A binary logistic regression analysis was performed with the subscale scores and composite scores of the shortened scale and respondent characteristics, namely nurses’ age, sex (male = 1, female = 0), and educational background (i.e., university or higher-level degree (=1) or not (=0)) as independent variables and the presence or absence of an intention to leave or remain as the dependent variable.

Reliability

To confirm internal consistency, Cronbach’s alpha coefficients were calculated for each subscale and composite to test the reliability of the instrument’s short form. Additionally, Pearson’s correlation coefficients were calculated for the seven factors. To determine whether each item in the shortened scale was an important component, we examined whether Cronbach's alpha coefficient decreased after removing the item from each subscale.

Ethical Approval

This study was approved by the Ethics Review Board of the organization to which the first author is affiliated (No. C2022-054). Participants were provided with information about the study's aim, and informed consent was obtained through a questionnaire.

## Results

Participant characteristics

The characteristics of the professionals in home healthcare who participated in the survey are presented in Table 1. Most participants were females (329, 86.6%) with an average age of 44.6 years (SD = 10.8). Three-quarters of the participants (288, 75.8%) worked full-time. The most common qualifications as a healthcare professional were registered nurse, 295 (77.6%); followed by physical therapist, 49 (12.9%); occupational therapist, 21 (5.5%); and others, 15 (4.0%). Among the healthcare professionals, 324 individuals (85.3%) had the intention to remain. On a scale of 0 to 100, their job satisfaction was 65.7 (SD = 25.1), and the quality of care provided by the agencies was 71.0 (SD = 20.2).

**Table 1:** Characteristics of participants in this study (n = 380) ^a^Samples with missing data were removed. SD, standard deviation

Individual-level variables		N or mean	(%) or SD
Sex (n, %)	Male	51	(13.4)
	Female	329	(86.6)
Age (n, %)	20s	39	(10.3)
	30s	83	(21.8)
	40s	125	(32.9)
	≥ 50	129	(33.9)
	Non-response	4	(1.1)
Academic background (n, %)	Bachelor's degree	61	(16.1)
	Graduate program	3	(0.8)
	Others (e.g., vocational school, associate's degree)	315	(82.8)
	Non-response	1	(0.3)
Employment status (n, %)	Full-time	288	(75.8)
	Part-time	88	(23.2)
	Non-response	4	(1.1)
Professions (n, %)	Registered nurses	295	(77.6)
	Licensed practical nurse	14	(3.7)
	Physical therapist	49	(12.9)
	Occupational therapist	21	(5.5)
	Speech-language-hearing therapists	1	(0.3)
Intention to remain/leave in the workplace (n, %)	Intention to leave	53	(13.9)
	Intention to remain	324	(85.3)
	Non-response	3	(0.8)
Age^a^ (n = 376)	(Mean, SD)	44.6	10.8
Profession experiences^a^ (n = 377)	(Mean, SD)	18.0	10.1
Work experiences at the current agency^a^ (n = 379)	(Mean, SD)	5.0	5.4
Job satisfaction^a^ (n = 374)	(Mean, SD)	65.7	25.1
Quality of care^a^ (n = 373)	(Mean, SD)	71.0	20.2

The founding entities of the agencies where participants worked were as follows: 144 for-profit corporations (37.9%), 124 medical corporations (32.6%), and 49 associations/foundations (12.9%). The number of full-time equivalent staff per agency was ≥ 10 staff: n = 156 (41.1%), seven to less than 10 staff: n = 85 (22.4%), five to less than seven staff: n = 68 (17.9%), and three to less than five staff: n = 62 (16.3%). The total number of monthly visits to client homes per agency was ≥ 501 visits: n = 144 (37.9%), 301-500 visits: n = 72 (18.9%), 201-300 visits: n = 46 (12.1%), 101-200 visits: n = 55 (14.5%), and ≤ 100 visits: n = 35 (9.2%).

Validity

Twenty-one items were retained to create the original instrument’s short-form (Table 2, Appendices), each was selected by three nursing researchers based on its ratings by three home healthcare expert nurses.

**Table 2:** Item details and scores for all items of PES-HHC (n = 380) ^a^Item numbers for the original 37-item scale. PES-HHC, Practice Environment Scale for Home Healthcare

Subscale		Item no.^a^	Item	Mean	SD
1	Manager ability, leadership, and support of healthcare professionals	1	My manager is a good manager and leader.	3.13	0.71
		5	Managers monitor the work of staff members and support them in order to develop their ability.	3.11	0.7
		6	Administrators consult with staff on daily problems and procedures.	3.11	0.7
2	Foundations for quality of care	9	Active staff development or continuing education programs for healthcare professionals.	2.57	0.8
		14	There are opportunities for all healthcare professional staff members, full-time and part-time, for learning how to provide high-quality care. (e.g., outside seminars, in-service training, case studies.)	2.91	0.69
		15	A clear philosophy of service that pervades the patient care environment.	2.68	0.69
3	Good relationships among multiple disciplines inside and outside of the organization	16	The work environment allows you to share your opinions or questions based on your healthcare professionals’ judgment with your colleagues (nurses or other disciplines), either directly or via another person.	3.18	0.57
		17	The work environment allows you to share your opinions or questions based on your healthcare professionals’ judgment with professionals in other agencies, either directly or via another person.	3.09	0.57
		18	The agency staff members consider mistakes as learning opportunities rather than occasions for blaming.	3.10	0.57
4	System for sharing the information required for care	22	There is a system for dealing with complaints from patients or family members in a timely manner.	3.03	0.6
		24	You can report your questions or concerns about your patient visits to your supervisor, and consult with her/him, on the same day they arise.	3.22	0.62
		25	Your agency can give you advice immediately when you contact them as you are having problems at a patient's house.	3.17	0.67
5	Personnel system that enables work-life balance	27	Your current workload allows you to have enough time for your private life or family.	2.89	0.75
		29	Your days off are assured	3.14	0.72
		30	Your work schedule is flexible and able to be adjusted in case of emergency.	3.05	0.75
6	Collegial relationship between healthcare professionals in the organization and physician	32	Collaboration (joint practice) between healthcare professionals and physicians.	2.89	0.57
		33	A lot of teamwork between healthcare professionals and physicians.	2.84	0.62
		34	Physicians and healthcare professionals have good working relationships.	2.85	0.58
7	Appropriate assignment of a case manager	35	A case manager who visits clients can have their assigned clients switched by mutual agreement with their supervisor as needed, considering their compatibility.	2.92	0.66
		36	A case manager who visits clients is assigned based on consideration of his or her experience, and strengths and weaknesses.	2.78	0.69
		37	A case manager who visits clients can have their assigned clients switched by mutual agreement with their supervisor as needed, based on aspects of the maintenance or improvement of the quality of care.	2.75	0.69

**Figure 3:**
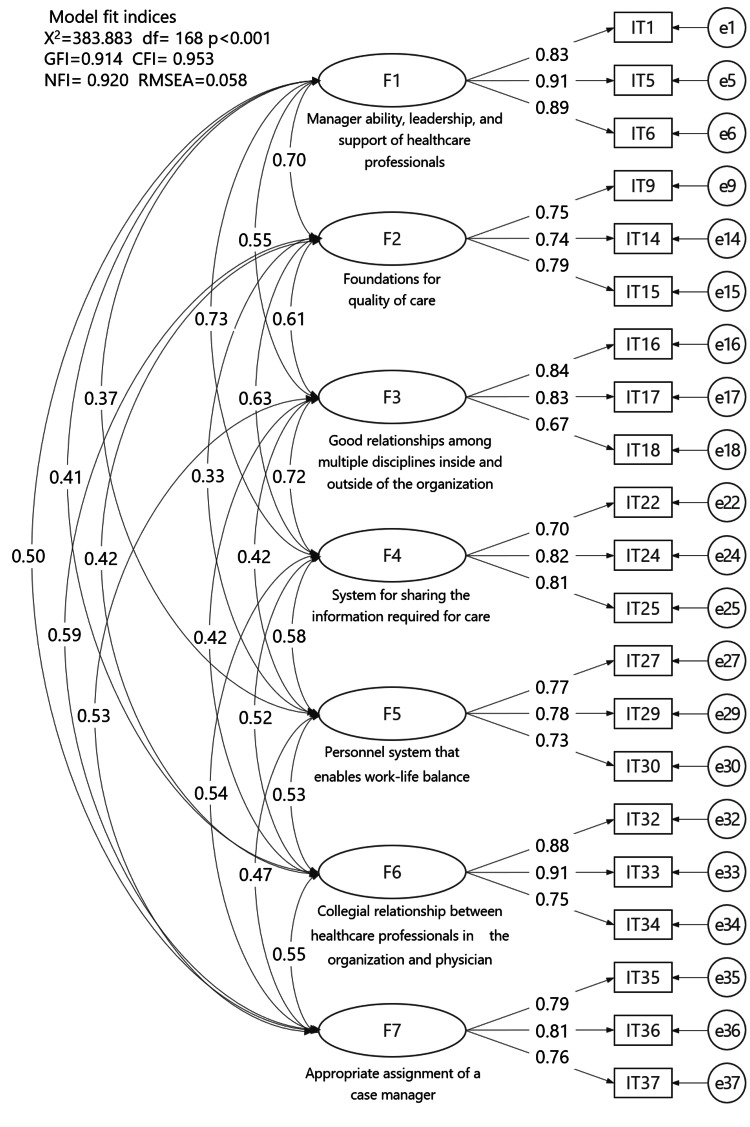
Confirmatory factor analysis of the PES-HHC (n = 380) CFI: Comparative Fit Index; df: degree of freedom; GFI: Goodness-of-Fit Index; NFI: Normed Fit Index; PES-HHC: Practice Environment Scale for Home Healthcare; RMSEA: root mean square error of approximation

Confirmatory factor analysis revealed that the 21-item PES-HHC had the same seven-factor structure as the original 37-item NPES-HHC (Figure 3). Model fit indices were χ² = 383.883 (degrees of freedom = 168, p < 0.001), GFI = 0.914, CFI = 0.953, NFI = 0.920, and RMSEA = 0.058. The seven subscales and their composite scores are shown in Table 3.

**Table 3:** Mean subscale scores and Pearson's correlation coefficients for the PES-HHC (n = 380) ^a^Pearson's correlation coefficient **p < 0.001 CI, confidence interval; PES-HHC, Practice Environment Scale for Home Healthcare; SD, standard deviation; Sub, subscale; -, not applicable

PES-HHC: subscales and composite		Mean	SD	Cronbach’s α	Correlation^a ^(95% CI)						
					Sub 1	Sub 2	Sub 3	Sub 4	Sub 5	Sub 6	Sub 7
Sub 1	Manager ability, leadership, and support of healthcare professionals	3.12	0.65	0.91	-	-	-	-	-	-	-
Sub 2	Foundations for quality of care	2.72	0.62	0.80	0.597** (0.529-0.658)	-	-	-	-	-	-
Sub 3	Good relationships among multiple disciplines inside and outside of the organization	3.12	0.49	0.81	0.519** (0.441-0.589)	0.536** (0.461-0.604)	-	-	-	-	-
Sub 4	System for sharing the information required for care	3.14	0.54	0.81	0.640** (0.577-0.696)	0.529** (0.452-0.598)	0.636** (0.572-0.692)	-	-	-	-
Sub 5	Personnel system that enables work-life balance	3.03	0.63	0.80	0.330** (0.237-0.417)	0.261** (0.165-0.353)	0.356** (0.265-0.441)	0.483** (0.402-0.557)	-	-	-
Sub 6	Collegial relationship between healthcare professionals in the organization and physician	2.86	0.53	0.88	0.388** (0.299-0.471)	0.378** (0.289-0.461)	0.396** (0.307-0.477)	0.486** (0.405-0.559)	0.459** (0.376-0.535)	-	-
Sub 7	Appropriate assignment of a case manager	2.82	0.59	0.83	0.429** (0.344-0.508)	0.475** (0.393-0.549)	0.458** (0.375-0.534)	0.466** (0.383-0.541)	0.397** (0.309-0.479)	0.491** (0.410-0.564)	-
Composite		2.97	0.42	0.85	0.770** (0.726-0.808)	0.742** (0.693-0.784)	0.743** (0.695-0.785)	0.819** (0.783-0.850)	0.646** (0.584-0.701)	0.691** (0.635-0.740)	0.725** (0.673-0.769)

On average, job satisfaction scored 65.7 (SD = 25.1), and quality of care was 71.0 (SD = 20.2) (Table 1). The partial correlation coefficients between job satisfaction, the seven subscales, and the composite ranged from 0.385 to 0.654 (Table 4). The partial correlation coefficients between quality of care, the seven subscales, and the composite ranged from 0.302 to 0.536 (Table 4).

**Table 4:** Relationship between PES-HHC, job satisfaction, quality of care, and intention to remain in the workplace ^a^Adjusted for age, sex, and educational background ^b^Evaluated by visual analog scale ^c^Dependent variable: Intention to remain (=1), intention to leave (=0) **p < 0.001 CI, confidence interval; PES-HHC, Practice Environment Scale for Home Healthcare; Sub, subscale

PES-HHC: subscales and composite		Partial correlation coefficients^a^ (n = 363)		Logistic regression analysis^a,c^ (n = 372)		
		Job satisfaction^b^	Quality of care^b^	Dependent variable: intention to remain in/leave the workplace		
				Odds ratio	95% CI	p-value
Sub 1	Manager ability, leadership, and support of healthcare professionals	0.562**	0.427**	1.823	1.168-2.844	0.008
Sub 2	Foundations for quality of care	0.500**	0.536**	2.476	1.482-4.136	0.001
Sub 3	Good relationships among multiple disciplines inside and outside of the organization	0.506**	0.378**	2.229	1.152-4.312	0.017
Sub 4	System for sharing the information required for care	0.515**	0.417**	1.960	1.128-3.407	0.017
Sub 5	Personnel system that enables work-life balance	0.495**	0.302**	2.09	1.321-3.306	0.002
Sub 6	Collegial relationship between healthcare professionals in the organization and physician	0.385**	0.361**	2.152	1.220-3.798	0.008
Sub 7	Appropriate assignment of a case manager	0.391**	0.323**	2.382	1.425-3.982	0.001
Composite		0.654**	0.534**	4.691	2.147-10.246	<0.001

With “intention to remain at the workplace” as the dependent variable and the seven subscales and PES-HHC composite as the independent variables, logistic regression analysis revealed that the odds ratios of the seven subscales and the composite were significantly positively related to “intention to remain at the organization” (Table 4). The odds ratios ranged from 1.823 for Subscale 1 to 4.691 for the composite.

Reliability

Cronbach alpha coefficients for the seven subscales’ scores ranged from 0.80 to 0.91 (Table 3); for the composite, the value was 0.85. For 18 of the 21 items, removing one item from each subscale resulted in a decrease in the alpha coefficient for each subscale. For the other three items, removing one item from each of the subscales 3, 4, and 6 resulted in an increase in the alpha coefficient for each subscale. However, because the increase in Cronbach's alpha coefficient after removing each item was very small (0.01-0.03), the three items were retained in their respective subscales. Pearson’s correlation coefficients for the PES-HHC subscales are presented in Table 3. The correlation coefficients between the seven subscales ranged from 0.261 to 0.640. All correlation coefficients were significant.

## Discussion

A reliable and valid shortened version of the PES-HHC was developed with the same seven-factor structure as the original scale by analyzing data from a cross-sectional survey of home healthcare agencies across Japan and verifying hypotheses (a) through (e). This shortened scale is characterized by three items per factor, as recommended in scale development as the absolute minimum number of items [23]. In addition, reflecting the current situation of healthcare professionals working in home healthcare agencies, the respondents included nurses and therapists (e.g., physical and occupational therapists). Implementing a reliable and valid shortened version of the scale will reduce the time and effort required for healthcare professionals to respond, thereby minimizing the burden on respondents and providing insights valuable to managers, researchers, and others interested in the results.

Reliability of the PES-HHC

The Cronbach’s alpha coefficients of the shortened version of the scale were similar to those of the original (shortened: 0.80-0.91, original: 0.77-0.92) [16]. Generally, the Cronbach’s alpha coefficient increases as the number of items increases. However, in this study, although the number of items decreased, the magnitude of the alpha coefficient was relatively unaffected. The closer the Cronbach’s alpha coefficient is to 1, the greater the internal consistency. An alpha coefficient of 0.7-0.8 is respectable, 0.8-0.9 is very good, and > 0.9 indicates that the number of items can be reduced [24]. In this study, although the number of questions comprising each factor was reduced to three items, the Cronbach's alpha coefficient exceeded 0.80, indicating high reliability. Therefore, hypothesis (a) was verified.

Validity of the PES-HHC

The PES-HHC, comprising seven factors and 21 items, was considered a valid scale due to its content validity, construct validity, and criterion-related validity.

Content validity is an important criterion according to the COSMIN checklist [25]. The selection of items in this study was based on an evaluation by three home healthcare expert nurses; items that adequately captured the content of the subscale were ranked higher. Hence, the items were selected based on the judgment of home healthcare expert nurses as to whether each item could measure the subscale. Finally, when deciding on the three items for each subscale, the three nursing researchers discussed whether the content was sufficient to measure the subscale based on the experts' judgment. From these results, it can be concluded that the shortened scale version was composed of items with content validity. From the stage of creating the original 37 items, an item pool was created to reflect the actual situation of home healthcare agencies [16]; the items clearly exhibit high content validity.

The results of the confirmatory factor analysis (Figure 3) showed that the PES-HHC, with 21 items, retains the same seven-factor structure as the original version, confirming the construct validity. The model fit indices of the confirmatory factor analysis in this study were comparable to those at the time of development [16], and the factor structure was maintained even after reducing the number of items; thus, hypothesis (b) was also supported.

Regarding the criterion-related validity, hypotheses (c), (d), and (e) were verified based on the positive relationship between the PES-HHC scores and job satisfaction, quality of care, and intention to remain at the current organization. Partial correlation coefficients between the subscale and composite and job satisfaction ranged from 0.33 to 0.61 for the original version [16] and from 0.39 to 0.65 for the shortened version (Table 4). Similarly, for quality of care, partial correlation coefficients ranged from 0.29 to 0.57 for the original 37-item scale [16] and from 0.30 to 0.54 for the 21-item scale (Table 4). The partial correlation coefficients in this study were similar to those used in the original version.

In a logistic regression analysis with intention to continue working as the dependent variable, odds ratios for the seven subscales and the composite score ranged from 2.7 to 18.4 in the original version [16] and from 1.8 to 4.7 in the shortened version. In the original version, the odds ratio for subscale 3 was 13.3, and for the composite variable was 18.4 [16], which were greater than the odds ratios in the present study (2.2 and 4.7, respectively). The difference in the magnitude of the association between the subscales and composite scores of the work environment scale and the respondents' intention to stay may be partially due to the respondents in this study being nurses and therapists. In addition, the original version was conducted before the coronavirus disease (COVID-19) pandemic, whereas the responses to this survey were conducted during the pandemic, which may have influenced the odds ratios. For instance, a survey of healthcare workers during the COVID-19 pandemic showed that the female sex is associated with an increased odds of intention to leave, and nurses had higher odds of intention to leave than most other health professionals [26]. Based on the magnitude and sign direction of the odds ratio, improvement of the work environment, as shown in the original and shortened versions, corresponds to a positive outcome of healthcare professionals’ intention-to-stay at the workplace.

Features of the shortened PES-HHC

Of the seven subscales in the shortened version, three were derived from the PES-NWI [9]. The names of the subscales have been revised to reflect the inclusion of therapists and other healthcare professionals as respondents; however, the essential characteristics of the items and factors remained unchanged. The following three factors are relevant: “Manager ability, leadership, and support of healthcare professionals (subscale 1),” “Foundations for quality of care (subscale 2),” and “Collegial relationship between healthcare professionals in the organization and physician (subscale 6).” Since the PES-NWI is related to healthcare quality, safety, and patient and clinician well-being [11], these subscales may also be related to such variables.

The other four subscales correspond to the original factors that measure the work environment in a home healthcare setting: “Good relationships among multiple disciplines inside and outside of the organization (subscale 3),” “System for sharing the information required for care (subscale 4),” “Personnel system that enables work-life balance (subscale 5),” and “Appropriate assignment of a case manager (subscale 7).” Hence, the shortened version comprises items collected from questionnaire surveys and interviews in a form that reflects the actual practice situation. These factors are potentially effective in attracting and retaining healthcare professionals. For instance, improving the home care work environment, such as “job resources,” including leadership, staffing, collaboration, and feedback, might enhance work engagement and reduce burnout [27]. Strategies to retain employees in home care include not only addressing dissatisfaction with pay and benefits but also implementing supportive and innovative approaches to schedules and teamwork, and working with staff to identify barriers and solutions [28]. Supportive and innovative approaches were similar to those presented in the shortened version of the subscale.

Limitations and future research implications

This study has certain limitations and strengths. First, this study did not evaluate reproducibility by comparing the results of two surveys conducted at specific intervals. However, since the Cronbach alpha coefficient was ≥ 0.80 for all subscales and composite scores, it can be concluded that the reliability of the shortened scale’s internal consistency was confirmed. Nevertheless, future studies should examine the reproducibility of the shortened scale version.

Second, the scope of the home healthcare workplace was limited in this study. Unpredictable situations encountered by healthcare workers in their clients’ homes, such as consumer harassment, were not included in the workplace environment. According to Grasmo et al. [29], difficult interactions, such as verbal abuse from consumers, are stressful for healthcare workers, and these environments are often unpredictable. Therefore, in this study, the practice environment scale focused on the organizational characteristics and human environment that enable stable responses to challenging situations, such as verbal abuse from consumers. However, among the subscales of the PES-HHC, factors related to dealing with unpredictable events at the patient's home, such as “System for sharing the information required for care” (subscale 4) and “Appropriate assignment of a case manager” (subscale 7), are included in this scale.

Third, both the shortened and original versions of this tool were developed based on the environment of home healthcare in Japan, including the Japanese LTC insurance system. As such, the generalizability of PES-HHC across different health care systems is limited. It is important for users to verify whether the conditions used in the scale items also apply to other countries. Furthermore, considering that the number of immigrant healthcare professionals in Japan may increase as the population declines, future research is necessary to investigate home healthcare work environments and the impact of foreign healthcare professionals. For instance, as migrants may have high job demands, limited social support, and stress related to acculturation [30], these aspects must be measured in work environments.

Fourth, the reduction of the number of items from 37 to 18 was based solely on the judgment of nurses and did not take into account the judgment of other healthcare professionals. However, our data was collected from respondents representing multiple professions, including nurses, physical therapists, occupational therapists, and speech-language-hearing therapists. Based on the analysis results of reliability and validity derived from a spectrum of professions, the outcomes could potentially be generalized across multiple professions.

Fifth, this study presents self-report bias. Data on patients’ verifications or data on actual retention rates were not derived. External validations with quality indicators, such as patient satisfaction or avoidance of unplanned emergency hospitalizations, could help address self-reported biases. Future research on correlations of PES-HHC with other non-self-reported data could confirm our study outcomes. More comparative studies using quality indicators and VAS-based quality of care, or studies of job satisfaction with other available scales, would confirm the validity of self-reported VAS scales.

Lastly, the participation rate and response rate were not high in this study. Low response rates can mean a high risk of selection bias or non-response bias. When interpreting the results, attention must be paid to these potential biases and limitations. With observational studies among real-world participants, there is no clear standard for an appropriate response rate to offset biases. Ethical considerations allowed voluntary participation, which meant including non-respondents. In this study, other than reporting an appropriate response rate, we considered that a sufficient number of responses could achieve the objectives of this study. This study ensured a sample size of at least seven times the number of items and at least 100 participants (n = 380), as recommended by the COSMIN Research Design Checklist [18]. In addition, to address potential biases, we randomly selected 2,000 home nursing care agencies by region to avoid bias based on participants' residential location, and confirmed the participants' willingness to participate in the study.

This study’s primary strength is the high Cronbach’s alpha coefficients, even with fewer items than the original version, confirming the same seven-factor structure as in the original version. Considering that it is a shortened version, the burden on the respondents is also low. Using this study to conduct a longitudinal analysis, it would be possible to verify whether work environment characteristics predict the actual turnover of healthcare professionals. These results will aid in developing strategies to attract and retain healthcare professionals in the LTC field.

## Conclusions

In our study, a reliable and valid 21-item shortened version of the PES-HHC was developed to measure an attractive practice environment for healthcare professionals. This provides several advantages: First, the low number of items will reduce the burden on healthcare professionals to participate, making it easier to use in busy practice settings. Second, since the characteristics of the work environment shown in the PES-HHC are related to the satisfaction and turnover intentions of healthcare professionals, improving the traits of the work environment in the PES-HHC will help to retain healthcare professionals in the organization. This will be particularly useful in home healthcare settings. However, it is worth noting that our study examined the relationship between the PES-HHC and intention-to-remain, but we did not cross-examine the results with the actual retention or actual turnover in respective workplaces. Future prospective research that examines the effect of PES-HHC on actual retention or turnover would be warranted.

## Appendices

**Table 5:** Revised short version of the Practice Environment Scale for Home Healthcare (PES-HHC) Each subscale score was calculated as the average of the responses to the questions on each subscale. The composite score was calculated as the average of the seven subscale scores. The seven subscales were as follows: 1. manager ability, leadership, and support of healthcare professionals (items 1, 2, and 3); 2. foundations for quality of care (items 4, 5, and 6); 3. good relationships among multiple disciplines inside and outside of the organization (items 7, 8, and 9); 4. system for sharing the information required for care (items 10, 11, and 12); 5. personnel system that enables work-life balance (items 13, 14, and 15); 6. collegial relationship between healthcare professionals in the organization and the physician (items 16, 17, and 18); and 7. appropriate assignment of a case manager (items 19, 20, and 21).

Practice Environment Scale for Home Healthcare (a revised short form)					
Instructions: For each item, please circle the appropriate number to indicate the extent to which you agree that the condition exists in your current workplace.					
Please answer from the perspective of either manager or staff. Managers should answer whether they are able to provide this to their staff. Healthcare professional staff include nurses, physical therapists, occupational therapists, speech-language-hearing therapists, etc.					
No.	Item	Strongly disagree	Disagree	Agree	Strongly agree
1	My manager is a good manager and leader.	1	2	3	4
2	Managers monitor the work of staff members and support them in order to develop their ability.	1	2	3	4
3	Administrators consult with staff on daily problems and procedures.	1	2	3	4
4	Active staff development or continuing education programs for healthcare professionals.	1	2	3	4
5	There are opportunities for all healthcare professional staff members, full-time and part-time, for learning how to provide high-quality care. (e.g., outside seminars, in-service training, case studies.)	1	2	3	4
6	A clear philosophy of service that pervades the patient care environment.	1	2	3	4
7	The work environment allows you to share your opinions or questions based on your healthcare professionals’ judgment with your colleagues (nurses or other disciplines), either directly or via another person.	1	2	3	4
8	The work environment allows you to share your opinions or questions based on your healthcare professionals’ judgment with professionals in other agencies, either directly or via another person.	1	2	3	4
9	The agency staff members consider mistakes as learning opportunities rather than occasions for blaming.	1	2	3	4
10	There is a system for dealing with complaints from patients or family members in a timely manner.	1	2	3	4
11	You can report your questions or concerns about your patient visits to your supervisor, and consult with her/him, on the same day they arise.	1	2	3	4
12	Your agency can give you advice immediately when you contact them as you are having problems at a patient's house.	1	2	3	4
13	Your current workload allows you to have enough time for your private life or family.	1	2	3	4
14	Your days off are assured	1	2	3	4
15	Your work schedule is flexible and able to be adjusted in case of emergency.	1	2	3	4
16	Collaboration (joint practice) between healthcare professionals and physicians.	1	2	3	4
17	A lot of teamwork between healthcare professionals and physicians.	1	2	3	4
18	Physicians and healthcare professionals have good working relationships.	1	2	3	4
19	A case manager who visits clients can have their assigned clients switched by mutual agreement with their supervisor as needed, considering their compatibility.	1	2	3	4
20	A case manager who visits clients is assigned based on consideration of his or her experience, and strengths and weaknesses.	1	2	3	4
21	A case manager who visits clients can have their assigned clients switched by mutual agreement with their supervisor as needed, based on aspects of the maintenance or improvement of the quality of care.	1	2	3	4
